# First continuous marine sponge cell line established

**DOI:** 10.1038/s41598-023-32394-x

**Published:** 2023-04-08

**Authors:** Kylie Hesp, Jans M. E. van der Heijden, Stephanie Munroe, Detmer Sipkema, Dirk E. Martens, Rene H. Wijffels, Shirley A. Pomponi

**Affiliations:** 1 Bioprocess Engineering, Wageningen University and Research, Wageningen, The Netherlands; 2 Harbor Branch Oceanographic Institute, Florida Atlantic University, Fort Pierce, FL USA; 3 Laboratory of Microbiology, Wageningen University and Research, Wageningen, The Netherlands; 4 Faculty of Biosciences and Aquaculture, Nord University, Bodø, Norway

**Keywords:** Biotechnology, Animal biotechnology, Cell culture

## Abstract

The potential of sponge-derived chemicals for pharmaceutical applications remains largely unexploited due to limited available biomass. Although many have attempted to culture marine sponge cells in vitro to create a scalable production platform for such biopharmaceuticals, these efforts have been mostly unsuccessful. We recently showed that *Geodia barretti* sponge cells could divide rapidly in M1 medium. In this study we established the first continuous marine sponge cell line, originating from *G. barretti*. *G. barretti* cells cultured in OpM1 medium, a modification of M1, grew more rapidly and to a higher density than in M1. Cells in OpM1 reached 1.74 population doublings after 30 min, more than twofold higher than the already rapid growth rate of 0.74 population doublings in 30 min in M1. The maximum number of population doublings increased from 5 doublings in M1 to at least 98 doublings in OpM1. Subcultured cells could be cryopreserved and used to inoculate new cultures. With these results, we have overcome a major obstacle that has blocked the path to producing biopharmaceuticals with sponge cells at industrial scale for decades.

Approximately 30% of the thousands of marine natural products discovered every year originate from sponges and sponge‐derived microbes^1–4^. Sponges also contain structural components with potential human health applications in tissue engineering, for example, as a scaffold to grow bone implants^5–7^. Many sponge‐derived products are potential drug candidates to treat human diseases^8–10^, such as cancer^11,12^ and infections with viruses^13^, fungi^14^ and drug-resistant bacteria^15^. For example, avarol, produced by the Mediterranean sponge *Dysidea avara*, has a strong cytostatic effect on leukemia cells^16,17^ and a recently discovered compound from a sponge-derived microbe prevents growth and biofilm formation of the bacterium *Staphylococcus epidermidis*, which poses a risk to patients who underwent surgery as it forms biofilms on implants and medical devices^18^. The model organism used in this study, *Geodia barretti*, produces barettins, a family of anti-inflammatory and antioxidant compounds^19^. For a comprehensive review of various types of bioactive compounds and their effects and structures, see Han et al. 2019^20^. Whilst their potential is enormous, sponge-derived drug candidates in various clinical trial stages face serious supply problems^21^. Wild harvest is unsustainable, and many compounds are too complex for chemical synthesis to be economically feasible^22^. Most efforts to overcome supply issues have thus focused on producing biomass using mariculture^22–25^ or cell culture^22,26–28^. Mariculture shows promise in some cases^24,25^, but culturing cells in vitro has major advantages: it allows close monitoring and control of conditions and is easy to optimize and scale up. Despite efforts of many groups, for a long time only primary cultures were established. Main obstacles in sponge cell culture were contaminating microbes and limited understanding of conditional requirements of sponge cells in vitro^26^.

Recently, we reported that cells of multiple sponge species could divide rapidly in M1^29^, a sponge-specific medium developed by Munroe et al. in 2019^30^. M1 medium is based on the defined M199 medium^30^, that was previously modified and used to set up primary cell cultures of various sponge species^27,28,31–33^. Amino acid concentrations were fine-tuned through multiple rounds of optimization using a genetic algorithm^30^. Proliferation in M1 medium was observed for cells from grass flat sponges *Geodia* sp. and *Amphimedon erina*, reef sponge *Geodia neptuni* and boreal deep-sea sponge *G. barretti*, among other species. Cultures of all 3 *Geodia* species divided rapidly until plateauing and could be passaged by subculturing up to 5 times and reach up to 7 population doublings (N_d_) depending on the species. This was the first concrete lead for developing stable marine sponge cell lines^29^. While these results represented a breakthrough, the number of passages and population doublings reached in M1 medium do not yield enough biomass to produce compounds at an industrial scale. To improve these growth characteristics of sponge cells in vitro, M1 medium was used as a base to optimize other components such as vitamins, fetal bovine serum and several growth factors (Table 1), using the same genetic algorithm that was used to develop M1 medium^30^. This resulted in a new medium composition, OpM1. Aiming to provide proof of concept that a sponge cell line could be scaled up to produce bioactive compounds in vitro, we tested whether *G. barretti* cells cultured in OpM1 medium had a higher growth rate, maximum cell density and maximum number of population doublings, due to the additional components.

**Table 1:** Compositions of M1 and OpM1 media.

	Ingredient	[x] stock	[x] M1*	[x] OpM1*	Unit	Cat. no
	M199 powder	–	9.1	9.1	g/L	M3769
Inorganic salts	NaCl	275	15.4203	15.4203	g/L	488,662
	Tris-HCl	50	3.7688	3.7688	g/L	T5941
	Tris-base	50	2.7838	2.7838	g/L	T1503
	MgCl_2_	200	10.0400	10.0400	g/L	M2393
	Na_2_SO_4_	50	0.8170	0.8170	g/L	238,597
	CaCl_2_	50	0.4000	0.4000	g/L	C7902
	KCl	50	0.3021	0.3021	g/L	P5405
Amino acids	L-Alanine	5	0.0281	0.0281	g/L	A3534
	L-Arginine	5	0.0250	0.0250	g/L	A6969
	L-Aspartic acid	5	0.0469	0.0469	g/L	A4534
	L-Asparagine	5	0.1031	0.1031	g/L	A0884
	L-Cysteine	5	0.0406	0.0406	g/L	C6852
	L-Glutamic acid	5	0.0469	0.0469	g/L	G5638
	L-Glutamine	5	0.0625	0.0880	g/L	G8540
	Glycine	5	0.0031	0.0031	g/L	G8790
	L-Histidine	5	0.1094	0.1094	g/L	H8125
	L-Isoleucine	5	0.0500	0.0500	g/L	I2752
	L-Leucine	5	0.0531	0.0531	g/L	L8912
	L-Lysine	5	0.0188	0.0188	g/L	L5626
	L-Methionine	5	0.0656	0.0656	g/L	M9625
	L-Phenylalanine	5	0.0938	0.0938	g/L	P5482
	L-Proline	5	0.0781	0.0781	g/L	P5607
	L-Serine	5	0.0844	0.0844	g/L	S5511
	L-Threonine	5	0.0250	0.0250	g/L	T8441
	L-Tryptophan	5	0.0719	0.0719	g/L	T0254
	L-Tyrosine	5	0.0469	0.0469	g/L	T1145
	L-Valine	5	0.1031	0.1031	g/L	V6504
AB/M**	Rifampicin	30	0.0300	0.0300	g/L	R3501
	Amphotericin B	0.2500	0.0025	0.0025	g/L	A2411
Vit1	Ascorbate · Na	0.5943	–	0.0015	g/L	A4034
	Pyruvate · Na	0.4400	–	0.0011	g/L	P5280
TE	Na_2_SiO_3_	1.4210	–	0.0018	g/L	307,815
	ZnSO_4_ · 7H_2_O	6.46E−02	–	0.0001	g/L	Z0251
GF1	Lectin from *Phaseolus vulgaris* (PHA)	4	–	4.72E−02	g/L	L1668
	EGF Recombinant Human Protein	1.60E−04	–	1.89E−06	g/L	10605HNAE50
	Insulin-like Growth Factor-1 Protein, Recombinant Human	8.00E−03	–	9.44E−05	g/L	GF-138
PFT α	Pifithrin-α	20	–	1.19E−02	g/L	P4359
PDGF	PDGF-BB Recombinant Mouse Protein	1.00E−01	–	8.69E−05	g/L	PMG0044
RPMI	RPMI 1640 Vitamin solution (100x)	–	–	3.11	µl/mL OpM1	R7256
FBS	Fetal Bovine Serum, qualified, One Shot™, US origin	–	–	4.35	µl/mL OpM1	A3160501
ITS-X	Insulin Transferrin Selenium Ethanolamine	–	–	11.18	µl/mL OpM1	51,500,056
LM-1	Lipid Mixture-1 (100x)	–	–	11.18	µl/mL OpM1	L0288

*Numbers in columns [x] M1 and [x] OpM1 do not include concentrations of nutrients present in M199 powder, only what is added to prepare both media.

**Antibiotics and antimycotics.

## Results

### OpM1 medium increases the maximum cell density of *G. barretti* in culture

**Figure 1:**
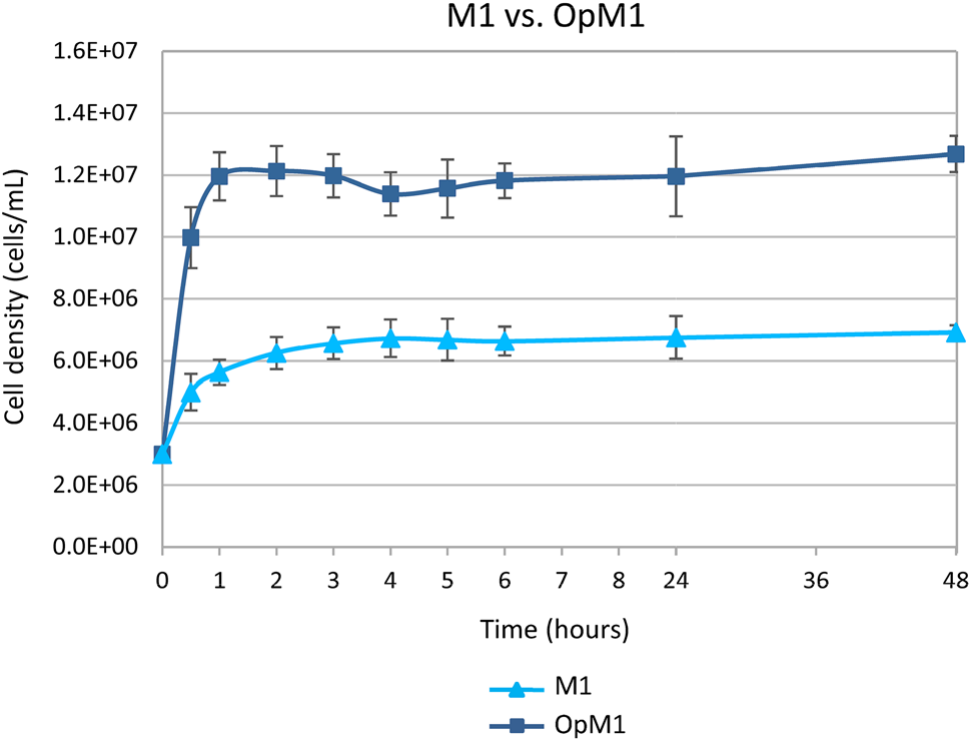
Growth curves of *G. barretti* cells of 3 individuals, cultured in M1 and OpM1 media. Error bars indicate the standard deviation from the average of biological (N = 3) and technical (n = 3) replicates.

To compare sponge cells proliferating in M1 and OpM1, growth of cells from 3 individuals of *G. barretti* was measured in both media (Fig. 1). These individuals were 3 different individuals from those used in our previous study^29^. Cells of 3 *G. barretti* individuals cultured in M1 reached 0.74 population doublings (N_d_) (from 3.0E+06 to 5.0E+06 ± 5.9E+05 cells/mL) after 30 min, where the cells cultured in OpM1 reached N_d_ = 1.74 (from 3.0E+06 to 1.0E+07 ± 9.9E+05 cells/mL) in the same period (Fig. 1). Cells of 3 *G. barretti* individuals in M1 reached a maximum density of 6.4E+06 ± 5.4E+05 cells/mL on average within 5 h after inoculation (Fig. 1), while cells in OpM1 medium reached a significantly (*p* = 1.6E−10) higher density of 1.2E+07 ± 2.3E+05 cells/mL after only 2 h (Fig. 1). After reaching their respective plateaus, the cells remained at the same density until the end of the culture (t = 48 h) in both media, with final cell densities averaging 6.9E+06 ± 1.2E+05 in M1 and a significantly higher value of 1.3E+07 ± 3.0E+05 cells/mL (*p* = 5.7E−14) in OpM1 (Fig. 1).

### Additional OpM1 components have varying impact on cell density

**Figure 2:**
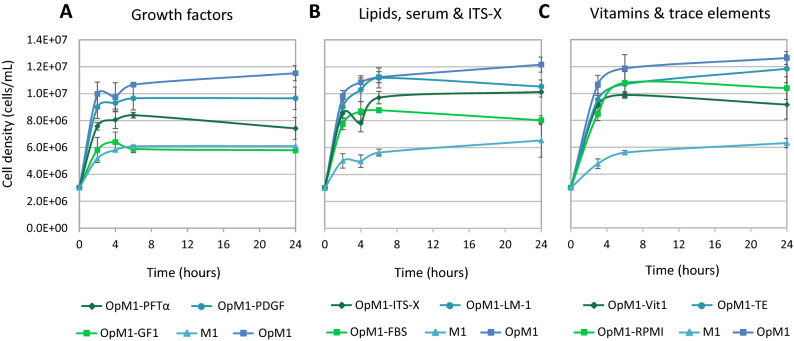
Impact of the additional components in OpM1 on the growth curve of *G. barretti* cells from 1 individual. Cells were cultured in different OpM1 media compositions, in each of which one component was replaced by sterile dH_2_O. (**A**) OpM1-PFTα, -PDGF, or -GF1, (**B**) OpM1- ITS-X, -LM-1 or -FBS, (**C**) OpM1-Vit1, -TE or -RPMI. OpM1 and M1 were used as controls. Error bars indicate the standard deviation from the average of technical replicates (n = 3).

OpM1 contains 13 additional components compared to M1 (see Table 1): growth factor cocktail 1 (GF1), platelet-derived growth factor (PDGF), pifithrin-α (PFTα), fetal bovine serum (FBS), insulin-transferrin-selenium-ethanolamine (ITS-X), lipid mixture-1 (LM-1), vitamin solution 1 (Vit1, consisting of sodium ascorbate (SA) and sodium pyruvate (SP)), trace elements solution (TE, consisting of sodium metasilicate and zinc sulfate), and Roswell Park Memorial Institute 1640 vitamin solution (RPMI). Effects of each component were assessed by comparing growth of cells from 1 individual of *G. barretti* in a set of media, in each of which a different component was absent, using OpM1 and M1 media as controls. The impact of leaving out the different components on the maximum cell density varied (Fig. 2).

Without GF1, which consists of epidermal growth factor (EGF), insulin-like growth factor-1 (IGF-1) and phytohemagglutinin (PHA) (Table 1), the growth curve was the same as in M1 medium (Fig. 2A). When PHA, IGF-1 and EGF were tested separately, all medium compositions without PHA performed like M1, while the composition with PHA showed no difference with the OpM1 control (Fig. S1A). The effect of PHA on cell density was dose-dependent: concentrations below 75% of the PHA concentration in OpM1 decreased the final density of *G. barretti* cells (Fig. S2A). Concentrations of PHA above 100% (up to 800%) did not increase the final cell density compared to OpM1 (Figure S2B). Absence of the small chemical apoptosis inhibitor PFTα^34^ or FBS lowered the cell density compared to the OpM1 control after 6 h, and after 24 h the density had decreased to the same level as in the M1 control (Fig. 2A,B). When Vit1 was omitted, the final density significantly decreased compared to OpM1 (Fig. 2C). Cells cultured in OpM1 without Vit1 but with either of its components, SA and SP (Table 1), reached a lower density than in OpM1 after 6 h, but after 24 h the difference with OpM1 was no longer significant (Figure S1B). In OpM1 without PDGF, LM-1, ITS-X or RPMI, a modest but significant decrease in cell density was observed (Fig. 2A–C). TE did not significantly affect the final density reached by *G. barretti* cells when omitted (Fig. 2C).

### *G. barretti* cells can reach at least 98 population doublings in OpM1 medium

**Figure 3:**
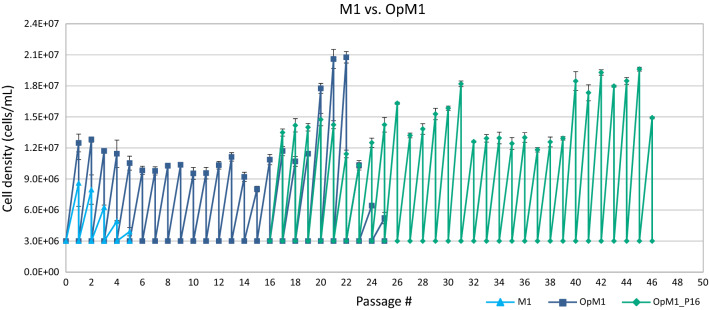
Passaging of *G. barretti* cells cultured in M1 and OpM1 media. OpM1_P16 represents cell cultures restarted from cells cryopreserved at passage 16. Error bars indicate the standard deviation from the average of biological (N = 3) and technical (n = 3) replicates.

**Figure 4:**
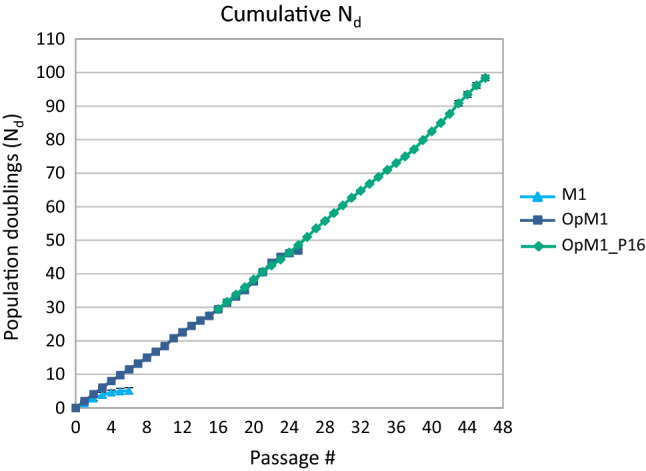
Cumulative population doublings (N_d_) of cells from 3 individuals of *G. barretti* cultured in M1 and OpM1 over 5 and 25 passages, respectively. OpM1_P16 shows doublings made by cells cryopreserved after passage 16 and used to start a new culture in OpM1, which continued for at least 30 more passages (46 passages total). Error bars indicate the standard deviation from the average of biological (N = 3) and technical (n = 3) replicates.

Subculture experiments were carried out in both M1 and OpM1 media at 4 °C to determine the maximum number of population doublings (N_dmax_) and passages that can be reached by cells of 3 *G. barretti* individuals. In M1 medium, cells subcultured twice a week divided until the 5th passage (P5) (Fig. 3). Cells of 3 *G. barretti* individuals cultured in M1 medium reached on average N_dmax_ = 5.1 ± 0.9 population doublings over 5 passages (Fig. 4). The N_d_ per passage was the highest in the first 3 passages and the cumulative N_d_ then plateaued, with only minor increases in cell number over the last 2 passages (Figs. 3 and 4). In contrast, for *G. barretti* cells cultured in OpM1, the N_d_ per passage was nearly constant at 1.8 ± 0.02 over 19 passages. After P19, cell densities increased for 3 passages, then decreased and cells stopped growing after 25 passages and N_d_ = 46.9 ± 0.7 (Fig. 3). Cells cryopreserved at the time of the 16th passage were used to inoculate OpM1 medium (OpM1_P16). This new culture was subcultured 30 times and reached N_d_ = 98.4 ± 0.8 (Fig. 4). The *G. barretti* cells in OpM1 continued to divide, and the N_dmax_ remains to be determined. At each passage, a portion of the cells was cryopreserved to create a cell bank for use in future experiments.

In the first 2 passages (P1, P2), densities reached among the cells of 3 *G. barretti* individuals cultured in M1 varied significantly. In P1, the cells of 1 individual (GB1) reached 5.9E + 06 ± 3.6E + 05 cells/mL, while the other 2 individuals reached higher cell densities of 1.0E+07 ± 6.6E+05 (GB2) and 8.1E+06 ± 5.1E+05 cells/mL (GB3), respectively. A similar pattern was observed in P2, where these individuals reached cell densities of 6.4E+06 ± 2.9E+05 (GB1), 9.8E+06 ± 4.6E+05 (GB2), and 7.7E+06 ± 4.5E+05 (GB3) cells/mL, respectively. Although this pattern of individual variation was not observed in P3 and P4, it occurred in P5. It should be noted that starting densities were calculated, rather than counted, by dividing the counted cell density by the dilution factor. This could also partially account for variations in starting and final cell densities between samples. Cells in OpM1 medium reached higher densities than those in M1 with little individual variation (Fig. 3).

### Cell line identity verified through 18S rRNA gene sequencing

**Figure 5:**
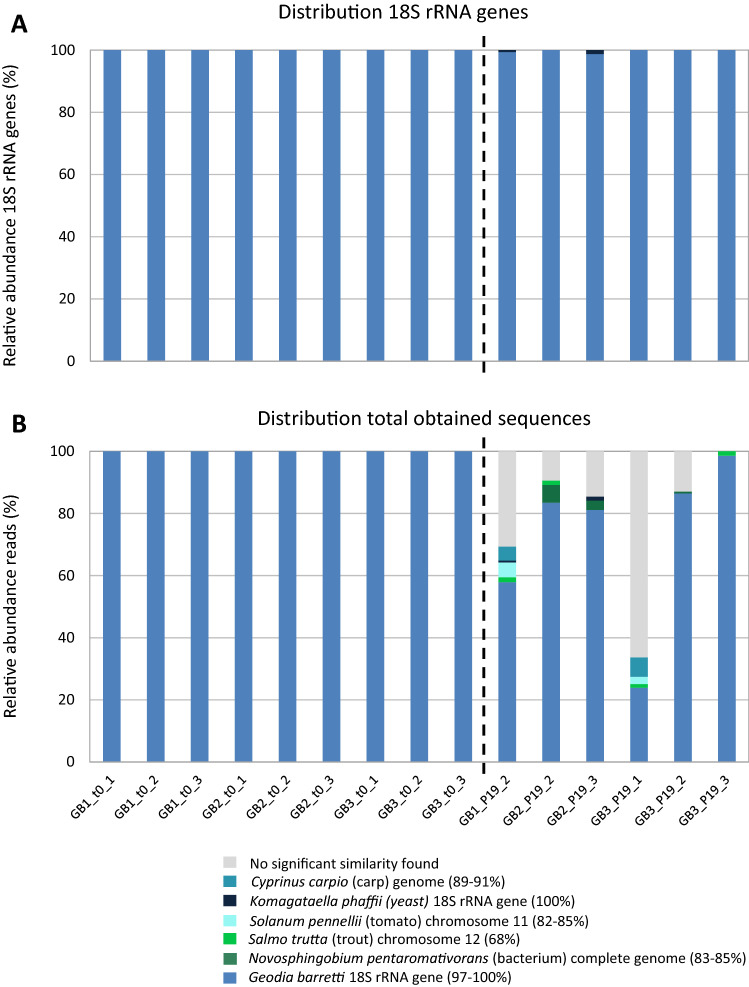
Eukaryotic community composition of *G. barretti* cell cultures. (**A**) Distribution of 18S rRNA genes in *G. barretti* cell cultures from the cell banks (t0) and after passage 19 (P19) separated by the dashed line. (**B**) Distribution of 18S rRNA gene sequences and other sequences that were generated from the cell banks (t0) and after passage 19 (P19). The color legend indicates the best Blastn hit obtained and in parentheses the percentage of identity.

18S rRNA gene sequencing was used prior to inoculation (P0) and after 19 passages (P19) to confirm that the cells growing in culture were *G. barretti* cells. At P0 all sequences had *Geodia barretti* voucher ZMBN:77922 18S rRNA gene (NCBI Genbank accession number KC481224.1) as best match (97–100% identity). Although three P19 samples were discarded due to a too low number of reads obtained (< 300), from each of the *G. barretti* specimens at least one P19 sample was successful. Two P19 samples, Gb1_P19_2 and Gb2_P19_3, contained a small fraction of *Komagataella phaffii* (yeast) cells, but this was less than 1.3%. All other reads identified as 18S rRNA in the P19 samples corresponded to *Geodia barretti* voucher ZMBN:77922 18S rRNA gene (97–100% identity) (Fig. 5A). DNA extraction yields were low for the P19 samples (30 ng/1.0E+08 cells on average) compared to the P0 samples (500 ng/1.0E+08 cells on average) that were directly extracted from the cell banks. Another noticeable difference between the P0 and P19 was that from the P19 samples an average of 22.3% of the reads (± 23.7%) obtained returned no blast hit or returned a hit that was no 18S rRNA gene (Fig. 5B). However, sequence identity for all reads with a non-18S rRNA gene match was below 92%; these were derived from fish (salmon, carp), tomato, and a bacterium (*Novospingobium pentaromativorans*). It is unclear what these sequences are derived from, but they may be related to media components containing DNA, such as FBS. However, there are no indications of substantial contamination of the *G. barretti* cell line with other eukaryotic cells at P19.

## Discussion

We established the first continuous marine sponge cell line, originating from the boreal deep-sea sponge *G. barretti*. We previously reported that cells from multiple sponge species, including *G. barretti*, divided rapidly in M1 medium and could be maintained for up to 5 passages and 7 population doublings^29^. In this study, we compared *G. barretti* cell growth in amino acid-optimized M1 medium^30^ and its derivative OpM1 medium that contains optimized concentrations of additional nutrients like growth factors, lipids, vitamins, and trace elements, among others (Table 1).

OpM1 significantly increased the growth rate and maximum cell density reached by *G. barretti* cells in culture, reducing the already short doubling time of less than 1 h in M1 medium to less than 30 min in OpM1. Doubling times in other animal cells are typically much longer, for example, Chinese hamster ovary (CHO) cells divide once per 20–24 h. However, sponge cells are known to proliferate rapidly in situ^35,36^: Within 6 h, 70.5% of all choanocytes in the sponge *Mycale microsigmatosa* had incorporated labelled bromodeoxyuridine (BrdU) while replicating their genome before dividing^35^. Sponge cells possess the same genes that regulate and drive cell cycling in all other animals^37^. Differences in these genes between sponges and other animals, and/or yet unknown genes or mechanisms, could have increased the speed of sponges’ replication machinery. It is also possible sponge cells do not need to replicate their genome before dividing when first placed in M1 or OpM1 medium. For example, *G. barretti* could possess pools of tetraploid cells (4 genome copies) that divide rapidly when the animal is damaged, as were found in other invertebrates, including the fresh‐water polyp *Hydra attenuata*^38^ and upside‐down jellyfish *Cassiopea* sp^39^. Such cell populations could be responsible for sponges’ potential to “heal” rapidly after mechanical damage^40,41^, although no significant pooling of tetraploid cells was observed in 2 freshwater sponge species^42^. Investigating how the *G. barretti* cell cycle progresses over multiple passages and how many genome copies cells have before and after dividing could shed light on how sponge cells proliferate so rapidly.

The maximum number of population doublings that could be reached by *G. barretti *cells increased from 5 doublings in M1 to at least 98 doublings in OpM1. Cells that were cryopreserved after the 16^th^ passage were used to inoculate new cultures that could be subcultured at least 30 more times, demonstrating that the established cell line can be stored in a cell bank for future research. The *G. barretti* cell line continued to divide in OpM1; research is currently ongoing in our group to determine whether the number of doublings the cell line can reach is limited, or whether the *G. barretti* cells are naturally immortal. In humans and most other animals, somatic cells can divide a limited number of times before they become senescent^43^. This so-called Hayflick limit lies between 40 and 60 divisions in human cells^44^ and is caused by shortening of the protective telomeres at the ends of chromosomes with each replication. Once a critical length is reached, the genome becomes instable and DNA damage response halts the cell cycle^45^. This mechanism stops uncontrollable cell division in multicellular organisms and is partly responsible for biological aging^43^. Sponges^46^ are among a number of marine invertebrate animals, including lobsters^47^, scallops^48^, and jellyfish^49^, known to maintain telomerase activity in their somatic cells, regardless of their age. Therefore, it would not be surprising to learn that the *G. barretti* cell line is naturally immortal. Comparing telomere length in cells from different passages could provide an answer to this question.

Our results show that all additions to OpM1, except the TE solution, EGF, and IGF-1, contributed to its superior performance compared to M1, but the impact of each component varied. PHA was paramount for the difference between OpM1 and M1, which is in line with previous reports of PHA-induced proliferation in cells of other marine sponges, *Axinella corrugata*^33^ and *Hymenidacidon heliophila*^27^. Higher PHA concentrations than in OpM1 did not further increase the cell density, suggesting other factors limit the maximum cell density in *G. barretti* cultures. Small chemical inhibitor of p53-mediated apoptosis^34^ PFTα and FBS also had a large impact, and removing these components reduced *G. barretti* cell growth similarly. After 6 h, *G. barretti* cells cultured in OpM1 without either component reached densities in between M1 and OpM1. After 24 h, the cell density had decreased to the same level as in M1. Therefore, we hypothesize PFTα and FBS together can prevent apoptosis in *G. barretti* cells. These results provide the first insights into specific nutrients and medium components required to maintain sponge cells in vitro. However, further testing is necessary to fully understand the effect of the different components in OpM1 on *G. barretti* cell growth and to learn how they affect the number of times *G. barretti* cells can divide.

Strong interspecies variation has been observed in sponges^29^ and it remains to be determined whether OpM1 medium can support cells of species other than *G. barretti* in long-term in vitro cultures. Each species will react differently to certain medium components and culture conditions to some degree, as illustrated by differences between cells from 12 different sponge species cultured in M1 medium in our previous work^29^. Additionally, both M1^30^ and OpM1 media were optimized for the Caribbean sponge *D. etheria* (unpublished data), in which it significantly increased metabolic activity but did not stimulate cells to divide. Individual variation needs to be considered when selecting source material^29,30,50^, even for species with low individual variation such as *G. barretti*^29^. Our results show that cells from the same individual can respond differently in separate experiments under the same culture conditions. While this could be because different medium batches and cryovials vary slightly, it would be beneficial to repeat (sub)culture experiments when screening sponge species and individuals. Another factor to consider is the presence and concentration of compounds of interest, or the sponge chemotype, which varies widely between species and also between individuals due to ecophysiological and seasonal factors^51,52^. In short, developing a production strain for any particular metabolite will require careful screening of source material, and species-specific optimizing of medium and culture conditions^29^.

In vitro sponge cell cultures can be used as model systems to test a wide variety of hypotheses about cell biology and how early animals evolved from unicellular organisms and developed symbiotic relationships with microbes. Metabolomics approaches can help identify pathways that synthesize secondary metabolites such as barettins. When such compounds are produced by the sponge itself, sponge cell lines can be used directly as a production platform. In cases where symbiotic microbes produce the compounds, sponge cell lines can be used to establish co-cultures with these microbes to create a production platform. The continuous *G. barretti* cell line presented here enables future steps to exploit the enormous potential of sponge-derived bioactive compounds.

## Conclusion

We established the first continuous marine sponge cell line, originating from the deep-sea boreal sponge *G. barretti*, by culturing cells in OpM1 medium. This derivative of M1 medium with added nutrients increased the growth rate, maximum cell density and maximum number of population doublings of *G. barretti* cells in vitro. PHA was the most important added component in OpM1, while PFTα and FBS also had large impacts. Our results may serve as a blueprint to establish cell lines for other sponge species. Interspecific and intraspecific (individual) variation makes careful screening of source material and species- and application-specific optimization of the culture medium essential to developing sponge cell lines. Cell lines for *G. barretti* and other sponge species can consistently produce enough biomass to develop small-scale production platforms and supply pharmaceutically relevant compounds for clinical trial studies. Producing biopharmaceuticals with sponge cells on an industrial scale is now one step closer to becoming a reality.

## Materials and methods

### Sample collection, dissociation and cryopreservation

Three individuals of *Geodia barretti* (Phylum Porifera, Class Demospongiae, Order Tetractinellida, Family Geodiidae) were collected from the ocean floor in a single trawl at a depth of ~ 500 m in a fjord close to Bergen, Norway (59°58.8″N 5°22.4″E). Cells were dissociated by squeezing fragments of the sponge through sterile gauze (B. Braun Medical, grade 16 mesh), and the resulting cell suspension was passed through a 40 μm filter to remove debris. Two wash steps followed, each consisting of centrifugation at 300 × *g* for 5 min and resuspension of the pellet in ASW. Cells were counted microscopically using hemocytometers (C-Chip™ Neubauer Improved, NanoEnTek) to determine cell concentrations. The cells were centrifuged once more at 300 × *g* for 5 min and resuspended in cryoprotectant (10% fetal bovine serum (FBS) and 10% dimethyl sulfoxide (DMSO) in ASW) at a density of approximately 1.0E + 08 cells/mL. Aliquots of 1 mL cell suspension in cryogenic vials were placed inside Nalgene® Mr. Frosty freezing containers, that cooled the cell suspension at a steady rate of 1 °C/minute when placed at −80 °C.

### Medium preparation

M1^30^ and OpM1 culture media compositions were as described in Table 1, where stock and final concentrations, manufacturer, and catalog number for each component can be found. Salt solutions added to M199 powder in dH_2_O resulted in a final osmolality and salt composition close to sea water (≈1000 mOsm). Next, stock solutions for each amino acid were added to reach the desired final concentration. Before the final addition of dH_2_O, antimicrobials (30 µg/mL rifampicin and 3 µg/mL amphotericin B) and additional OpM1 ingredients, aliquots of the medium were stored at -20 °C for up to 1 month. When medium was required, a thawed aliquot was either supplemented with dH_2_O and antimicrobials to make M1 medium, or with dH_2_O, antimicrobials and the other ingredients required to make OpM1. The final pH of both media was ≈8.2, similar to the pH of sea water. The concentrations of components in M1 and OpM1 during medium preparation were adjusted to account for dilution by addition of the inoculum, dH_2_O and antimicrobials. The inoculum always made up 1/16 of the total culture volume and contained 16 × the inoculation density (3.0E + 06 cells/mL): 4.8E + 07 cells/mL in ASW.

### Growth characterization

To characterize the growth of *G. barretti* cells in M1 and OpM1 media, cells from three individuals of *G. barretti* were cultured at 4 °C for 2 days (t = 48 h). Cryopreserved cells of each individual were thawed rapidly in a water bath set to 50 °C, then transferred to 1.5 mL Eppendorf tubes. The cells were washed twice in ASW by centrifugation at 300 × *g* for 5 min, removal of the supernatant and subsequent resuspension in 1 mL ASW. Cell concentrations were obtained using disposable hemocytometers (C-Chip™ Neubauer Improved, NanoEnTek). The cell suspension used to inoculate the culture was 1/16 of the final culture volume and always contained 4.8E + 07 cells/mL in ASW, to obtain a starting cell density of 3.0E + 06 cells/mL. The dilution of medium components by addition of the inoculum was considered during medium preparation, so that the final concentration of each component was as described in Table 1 after addition of the inoculum. The cells were microscopically counted every hour for the first 6 h, then after 24 and 48 h. From one constantly mixed cell suspension, triplicates of 250 µL cultures (≈ 3.0 + E06 cells/mL) in 48-well plates (CELLSTAR®, Greiner Bio-one, Cat. No. 677180) were prepared for each time point (8 time points per individual).

### Subculturing *G. barretti* cells

Cells of three *G. barretti* individuals were cultured at 4 °C in triplicate in 48-well plates (CELLSTAR®, Greiner Bio-one, Cat. No. 677180), with 250 µL cell suspension per well and a seeding density of 3.0 + E06 cells/mL. Cultures were passaged every 3 or 4 days. Cells that adhered to the bottom of the well were resuspended in the spent medium by pipetting up and down, then cell concentrations were determined microscopically, as described above. Subsequently, part of the cell suspension was mixed with fresh M1 or OpM1 medium to dilute back to the starting density in a total volume of 250 µL per well. This was continued until the cells were no longer dividing to determine the maximum number of population doublings of the cultures in each medium.

#### *G. barretti* cell bank

Cells remaining after subculturing in OpM1 were cryopreserved to create a cell bank for each passage. The method described in the section ‘Sample collection, dissociation & cryopreservation’ was used, except that OpM1 was used instead of ASW in the cryoprotectant (with 10% FBS and 10% DMSO) and the cell density was lower, around 1.0E + 07 cells/mL. To start a new culture from the cryopreserved cells in the cell bank, cells of 1 *G. barretti* individual were thawed and washed twice with ASW, following the protocol described in ‘Growth characterization’. Cells were then inoculated in triplicate in 48-well plates (CELLSTAR®, Greiner Bio-one, Cat. No. 677180), with 250 µL cell suspension per well and a seeding density of 3.0 + E06 cells/mL, and subcultured following the method described above.

### Eukaryotic community composition profiling

To verify the identity of the cells in culture, eukaryotic community composition was assessed from cell cultures from the three *G. barretti* individuals after passage 19, each cell culture in triplicate. In addition, eukaryotic community composition from triplicate cell bank samples from the same individuals were characterized. DNA was extracted from all samples using the FastDNA SPIN kit for soil (MP Biomedicals, Irvina, CA, USA) following the manufacturer’s instructions and using a pellet obtained by centrifuging cell suspension containing 1E8 cells (based on cell counts) as starting material. The concentration of the extracted DNA was determined with a Nanodrop 1000 spectrophotometer (Nanodrop Technologies, Wilmington, DE, USA), and its integrity was examined by gel electrophoresis on a 1% (w/v) agarose gel. The extracted DNA was dissolved in TE buffer and stored at -20 °C until PCR amplification. Amplification of v7-v8 region of the 18S rRNA genes was done according to Naim et al.^53^ with the modification that a 2-step PCR approach was applied. Briefly, in the first PCR step primers FF390 (CGATAACGAACGAGACCT) and FR1 (ANCCATTCAATCGGTANT)^54^ were were added to the 3’ end of Unitag1 and Unitag2, respectively^55^. The first PCR step was performed as in Naim et al., but with 25 instead of 30 cycles of amplification^53^. Subsequently, the first PCR product was used as template in a second PCR in order to add sample specific barcodes. The second PCR (5 cycles of amplification), PCR clean-up, and preparation of an equimolar mix of all samples was performed as described by Dat et al.^55^. The samples were sequenced on an Illumina HiSeq platform at Novogene (Cambridge, UK).

Illumina sequencing data were processed and analyzed using the NG-Tax pipeline^56^. Briefly, paired-end libraries were combined, and only read pairs with perfectly matching primers and barcodes were retained. To this end, both primers were barcoded to facilitate identification of chimeras produced during library generation after pooling of individual PCR products. Both forward and reverse reads were trimmed to 100 bp and concatenated to yield sequences of 200 bp. Demultiplexing, amplicon sequence variant (ASV) picking, chimera removal and taxonomic assignment were performed within one single step using the ASV_picking_pair_end_read script in NG-Tax. Reads were ranked per sample by abundance and ASVs (at a 100% identity level) were added to an initial ASV table for that sample starting from the most abundant sequence until the abundance was lower than 0.1%. The final ASV table was created by clustering the reads that were initially discarded as they represented ASVs < 0.1% of the relative abundance with the ASVs from the initial ASV table with a threshold of (98.5% similarity). Samples with < 300 reads were removed from the analysis and for all ASVs with an overall relative abundance > 0.2% and ASVs with a relative abundance > 1% in at least one sample were taxonomy assigned was by Blasting (Blastn) the ASVs against the nr/nt NCBI database on March 7, 2021. Hits were sorted based on the highest total score.

## Supplementary Information

Supplementary Information.

## Data Availability

Sequencing data were deposited in the NCBI Sequence Read Archive under BioProject ID: PRJNA765353 with accession numbers: SAMN21554876-SAMN21554890. Other datasets generated and/or analyzed during the current study are available from the corresponding author upon reasonable request.
