# Supplementary material for: First continuous marine sponge cell line established
Source: Sci Rep. 2023 Apr 8;13:5766. doi: 10.1038/s41598-023-32394-x (PMC10082835; doi:10.1038/s41598-023-32394-x)
Supplement: Supplementary file 1 — Supplementary Information. [file 41598_2023_32394_MOESM1_ESM.pdf]

Supplemental Materials

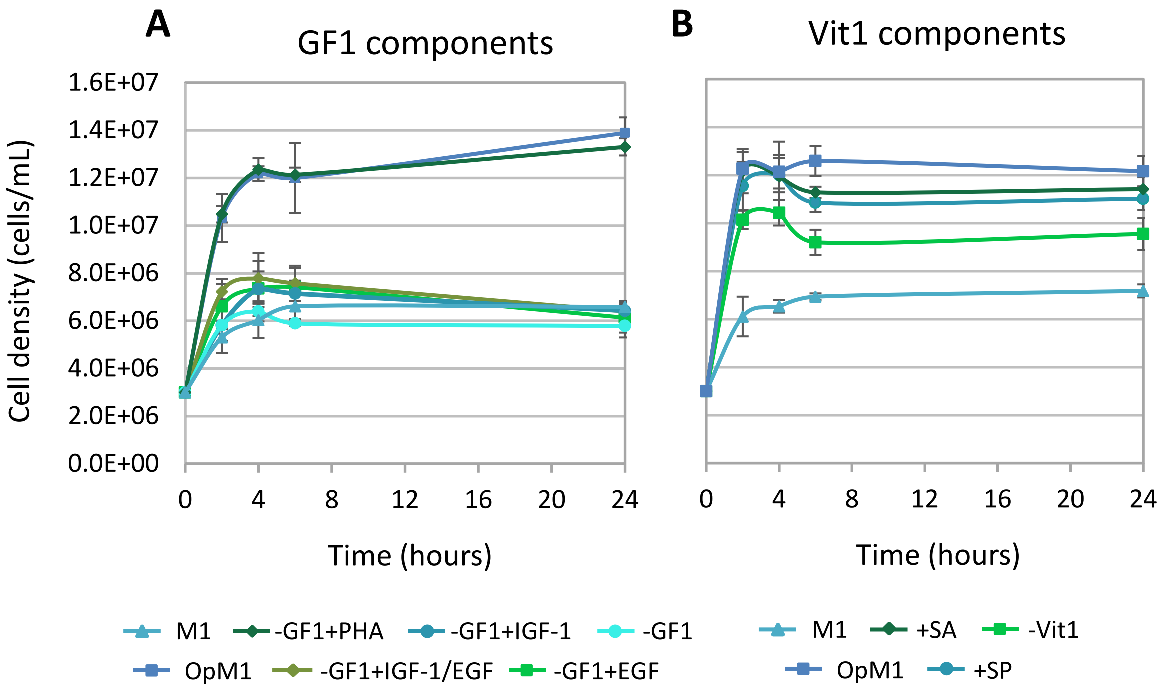

Figure S1. *Growth curves of cells from 1 G. barretti individual show the impact of individual ingredients of* ***A.*** *GF1: Cells cultured in OpM1-GF1+PHA, +IGF-1, +EGF or +IGF-1/EGF compared to cells cultured in OpM1-GF1,* ***B.*** *Vit1: Cells cultured in OpM1-Vit1, +SA or +SP compared to cells cultured in OpM1-Vit1. OpM1 and M1 were used as controls. Error bars indicate the standard deviation from the average of technical replicates (n=3).*

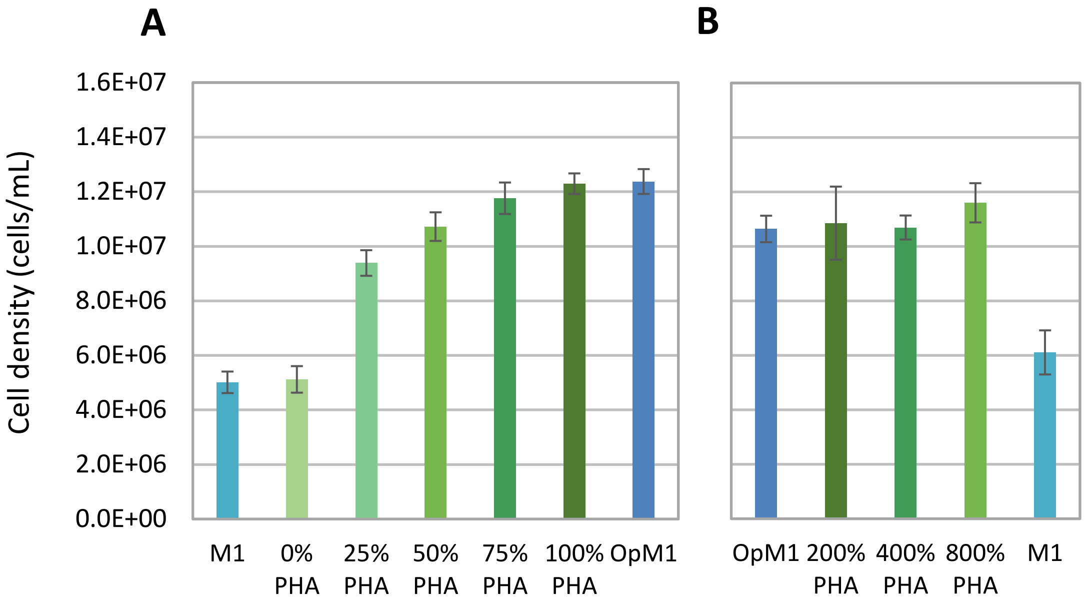

Figure S2. *Dose-dependency of the final cell density reached by cells of 1 G. barretti individual on PHA concentration in OpM1 medium. OpM1-GF1 with* ***A.*** *0, 25, 50, 75 and 100%, and* ***B.*** *200, 400 and 800% of the PHA concentration in OpM1. OpM1 and M1 were used as controls in both experiments. Error bars indicate the standard deviation from the average of technical replicates (n=3).*
